# CD22 Expression in B-Cell Acute Lymphoblastic Leukemia: Biological Significance and Implications for Inotuzumab Therapy in Adults

**DOI:** 10.3390/cancers12020303

**Published:** 2020-01-28

**Authors:** Francesco Lanza, Enrico Maffini, Michela Rondoni, Evita Massari, Angelo Corso Faini, Fabio Malavasi

**Affiliations:** 1 Hematology Unit & Romagna Transplant Network, Ravenna Hospital, 48121 Ravenna, Italy; enrico.maffini@auslromagna.it (E.M.); michela.rondoni@auslromagna.it (M.R.); 2 Clinical Pathology Unit, Hub Laboratory, Romagna Transplant Network, 47522 Cesena (FC), Italy; evita.massari@auslromagna.it; 3 Department of Medical Science, University of Torino and Fondazione Ricerca Molinette, 10126 Torino, Italy; angelo.faini@edu.unito.it (A.C.F.); fabio.malavasi@unito.it (F.M.)

**Keywords:** B-ALL, CD22, inotuzumab, antigen modulation

## Abstract

CD22 is a surface molecule expressed early during the ontogeny of B cells in the bone marrow and spleen, and can be found on B cells isolated from the different lymphoid compartments in humans. CD22 is expressed by most blasts from the majority (60–90%) of B-cell acute lymphoblastic leukemia (B-ALL). Current therapies in adults with newly diagnosed B-ALL are associated with complete remission (CR) rates of 50–90%. However, 30–60% of these patients relapse, and only 25–40% achieve disease-free survival of three years or more. Chemotherapy regimens for patients with refractory/relapsed B-ALL are associated with CR rates ranging from 31% to 44%. Novel immune-targeted therapies, such as blinatumomab and inotuzumab (a humanized anti-CD22 monoclonal antibody conjugated to the cytotoxic antibiotic agent calicheamicin), provide potential means of circumventing chemo-refractory B-ALL cells through novel mechanisms of action. Eighty percent of inotuzumab-treated B-ALL patients may achieve a CR state. This review is focused on the biological and clinical activities of CD22 antibodies in B-ALL, and provides evidence about the potential role played by qualitative and quantitative analysis of the CD22 molecule on individual B-ALL blasts in predicting the depletion of leukemic cells, and, ultimately, leading to better clinical response rates.

## 1. B-Cell Acute Lymphoblastic Leukemia 

**Figure 1:**
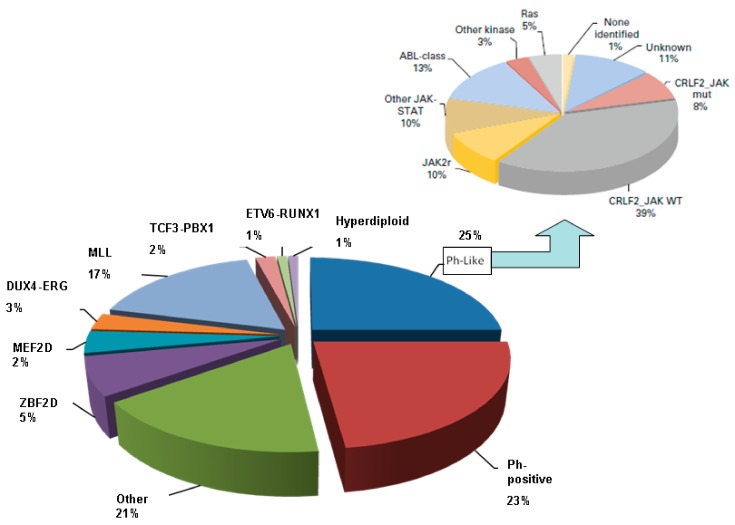
Genetic landscape of adult B-cell acute lymphoblastic leukemia.

A rare disease in adults, B-cell type acute lymphoblastic leukemia (ALL), is the most common form of acute leukemia among children, making up about 80% of diagnoses. Adult ALL at diagnosis tends to be higher risk, and approximately 20% of adult patients experience unfavorable outcomes. After an initial period of complete remission, adult patients typically develop chemoresistance and disease relapse [1,2]. Since this contrasts so strongly with the impressive rate of total remission in pediatric patients, hematologists have begun using drugs from pediatric therapeutic protocols for adult patients as well. Molecular and cytogenetic features determined at diagnosis are used to classify the B-ALL subtype and predict the risk for relapse. Nowadays, the application of molecular studies has shown that B-ALL is a highly heterogeneous disease at the genetic level; some of these mutations may result in the activation of aberrant pathways and influence cell survival [3]. Chromosome and genetic studies have demonstrated that t(12;21)/*ETV6-RUNX1* and high hyper-diploidy are good-risk prognostic features, whereas t(4;11)/*KMT2A* (*MLL*) translocations, t(17;19)/*TCF3-HLF*, low hypodiploid, complex cytogenetics (>5 chromosomal abnormalities) are high-risk characteristics. Moreover, cytogenetic analysis helps identify patients with Ph-positive t(9;22)/*BCR-ABL1* or other chromosomal alterations with prognostic relevance (Burkitt karyotypes). Recent studies reported a subgroup of B-ALL patients characterized by a gene expression profile similar to *BCR-ABL1* positive ALL. Although these patients lacked the fusion gene they shared the same poor outcome. This disease category was termed *BCR-ABL1*-like, and it is essentially heterogeneous: a deletion mutations in the IKZF1 gene encoding the transcription factor IKAROS underlie many cases; *CRLF2* rearrangements and *JAK2* mutations are detectable in a significant proportion of patients with a PH-like signature. This entity represents 10% of ALL cases in children, and 25–30% in adults. High-risk genetic biomarkers are four times more frequent in adults compared to children, whereas genetic biomarkers associated with a good prognosis account for 60% of pediatric and adolescent ALL but 15% of adult ALL, with ETV6-RUNX1 being virtually absent in adults aged over 30 years [4] (Figure 1).

**Figure 2:**
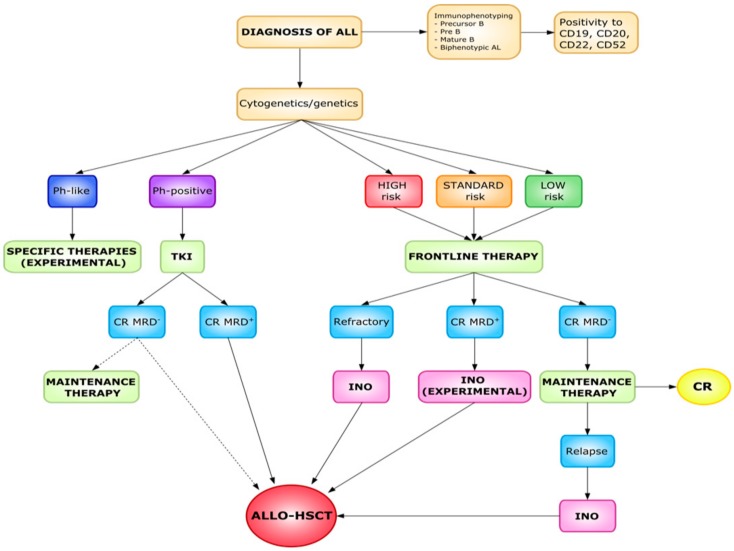
Overview of adult B-cell acute lymphoblastic leukemia treatments.

Current therapies in adults with newly diagnosed B-cell ALL are associated with complete remission (CR) rates of 50–90%. However, 30–60% of these patients eventually relapse, and only 25–40% achieve disease-free survival of three years or more. Patients affected by relapsed/refractory (R/R) disease have poor outcomes: fewer than 5–10% are expected to survive for at least five years from diagnosis. Current standard of care (SC) regimens (fludarabine, idarubicin, and high dose cytarabine-FLAG; cytarabine plus mitoxantrone; high dose cytarabine, among others) for adults with R/R B-cell ALL are associated with CR rates ranging from 31% to 44% during first salvage therapy. This falls to 18–25% during second salvage therapy. The majority of adult patients suffering from Philadelphia-negative (Ph-neg) B-ALL generally relapse after an initial response, while approximately 20% will have primary resistant disease. Allogeneic hematopoietic stem cell transplantation (HSCT) is still the only curative option available, but is only applicable to a minority of patients and produces better results as a form of consolidation therapy in the first-line setting. Novel immune-targeted therapies (e.g., drugs targeting the B cell-associated antigens CD19 (blinatumomab) and CD22 (inotuzumab) provide a potential means of circumventing chemo-refractory B-ALL cells through novel mechanisms of action and of preventing minimal residual disease (MRD). They make it possible for MRD-negative patients to receive allogeneic HSCT, significantly improving their clinical outcomes [5,6]. Targeted approaches, such as these have begun to transform the way we care for patients with resistant/relapsing (R/R) B-ALL [7] (Figure 2).

We focused our attention on the biological and clinical activity of CD22 antibodies in B-ALL, providing evidence about the potential role played by CD22 receptors analysis on B-ALL blasts in predicting leukemic cell killing, and, ultimately, in better clinical response rates.

## 2. CD22 Molecule and Function 

**Figure 3:**
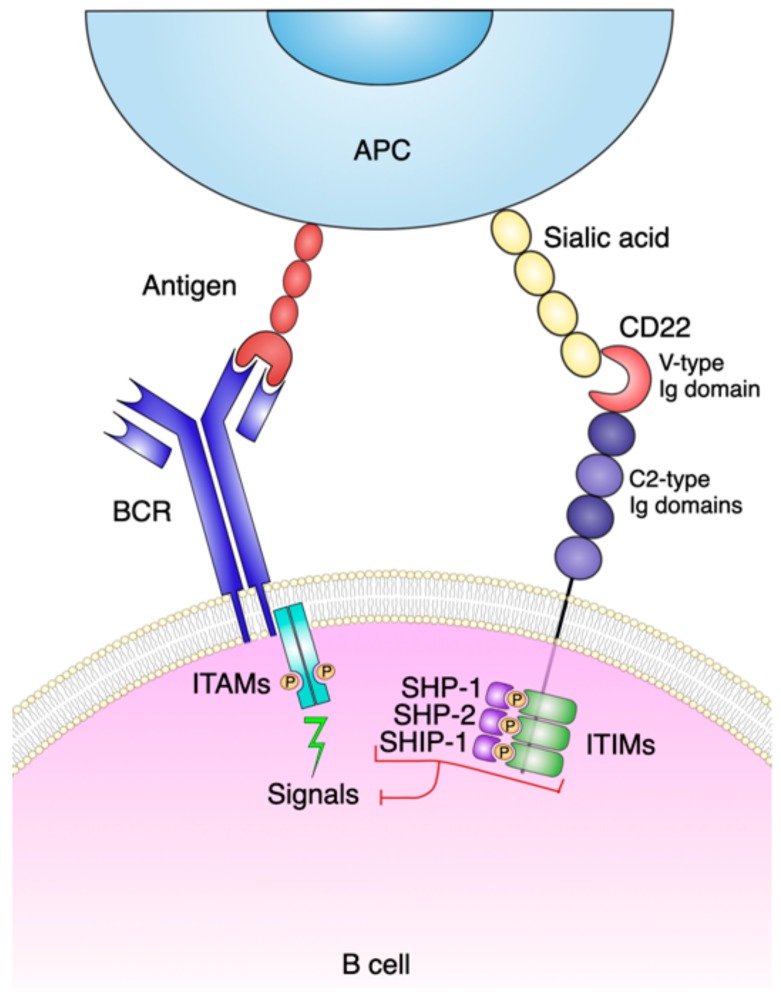
Structure, interactions, and biological activities of human CD22. The structure of the molecule includes an extracellular domain (six C-type and one V-type immunoglobulin domain). The intracellular domain encompasses immunoreceptor tyrosine-based inhibitory motifs (ITIMs). When sialic acids expressed by antigen presenting cells (APC) bind to CD22, the tyrosine residues of the ITIMs are phosphorylated. Ligation of the phosphorylated ITIMs to Src homology region 2 domain-containing phosphatase-1 and -2 (SHP-1 and SHP-2) and to Src homology region 2 domain-containing inositol phosphatase-1 (SHIP-1) induces a down-regulation of BCR-mediated signaling.

CD22 is expressed during the early stages of ontogeny of B cells in the bone marrow (BM) and spleen. It is also expressed by B lymphocytes isolated from the different lymphoid compartments. The molecule is up-regulated on the cell surface of activated B-lymphocytes, and is found in the cytoplasm of precursor B-cells (pro-B and pre-B lymphocytes) [8]. Sharing some general steps of the mechanistic functioning of the T cell receptor (TCR), the activation of B lymphocytes requires tight coordination, which results from the actions of multiple activating and inhibitory surface receptors. The key actor is the B cell receptor (BCR), flanked by the proteins of the sialic acid binding immunoglobulin-like lectin (Siglec) family. In brief, BCR provides the specificity of action, while the Siglec family contributes to the regulation of the signals implemented by BCR [9]. The Siglec family is a group of transmembrane proteins which share multiple structural features. Their extracellular portion consists of a variety of immunoglobulin (Ig) domains with a conserved N-terminal V-set Ig ligand binding sequence. The transmembrane and cytoplasmic regions are equipped with signaling motifs, primarily based on immunoreceptor tyrosine-based inhibition (ITIM). Sialic acids attached to the carbohydrate structures of glycans are the ligands of Siglec proteins. Different Siglecs are characterized by a preferential binding to sialic acids in distinct lineages [10]. Two Siglec proteins are present on B lymphocytes, namely CD22 (Siglec-2) and Siglec-G; both contribute to the negative modulation of BCR signaling [11,12]. CD22 expression is restricted to B lymphocytes. The ITIMs in their cytoplasmic tails are involved in the recruitment of tyrosine phosphatase SHP-1, which is followed by an inhibition of BCR-induced signals [13]. Its main functions in murine CD22 knock-out models is prevalent on conventional B cells (also called B2-cells) [14]. In contrast, Siglec-G has a wider tissue distribution, which includes B lymphocytes, dendritic cells and eosinophils. Further, Siglec-G inhibits BCR signaling on the B1-a cell population in murine models [15]. Both Siglec proteins on B lymphocytes show the binding of sialic acids typical of the family. CD22 binds α2,6-linked sialic acids (α2,6Sia), while Siglec-G interacts with α2,6- as well as with α2,3-linked sialic acids. These observations suggest that CD22 could be an adhesion molecule belonging to the Ig super family. Its extra-cellular portion is composed of seven Ig-like domains, the most distal of which recognizes glycol-conjugates containing α2,6Sia. α2,6Sia is present at the terminal of N-linked glycans and is expressed by different cells, including erythrocytes, monocytes, as well as T and B lymphocytes. α2,6Sia is also detectable on soluble plasma proteins, such as serum soluble IgM (sIgM) [16]. What emerges is a comprehensive model where CD22 and Siglec-G act as co-receptors engaged in the negative fine-tuning of BCR signaling mediated by continuous crosstalk with different cells. A characteristic feature of the Siglec molecules is their cell surface localization: both members of the family are located in discrete membrane micro domains proximal to the BCR. Their organization and dynamics on the membrane are orchestrated to different extents through the interaction with their ligands (Figure 3). In virtue of its restricted expression and intrinsic functional properties, CD22 was adopted as a target for antibody therapy of selected B cell leukemias [7].

## 3. In Vivo Targeting of Human CD22

**Figure 4:**
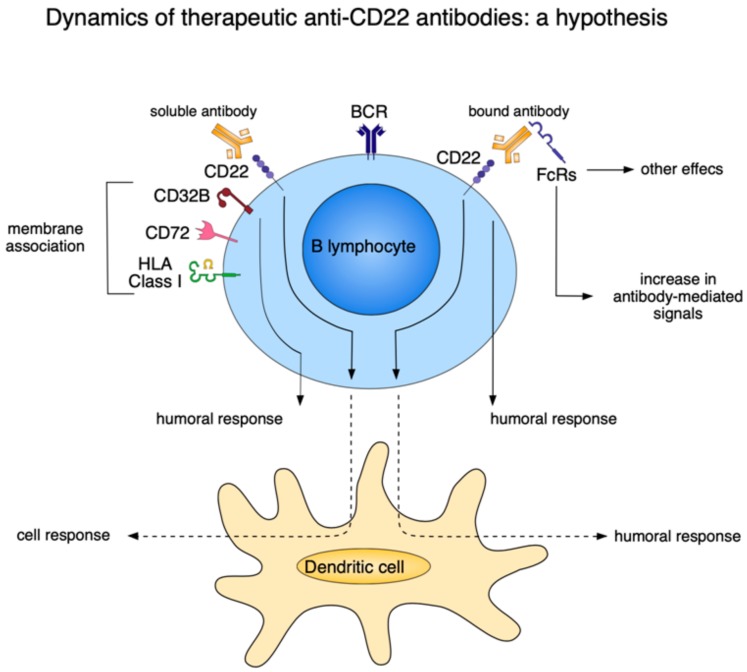
Hypothetical view of the effects induced by the interaction of CD22 with specific antibodies. CD22 can be bound by soluble antibodies as well as by FcR-insolubilized antibodies. When CD22 ligation by a soluble antibody occurs, the interaction with other molecules closely located on the same membrane micro domain (CD32B, CD72, and HLA Class I, among others) may induce humoral as well as a dendritic cell-mediated responses. When CD22 is bound by the insolubilized antibody, this lead to an increase of antibody-mediated signals on the FcR+ cells. A consequence is that CD22 might be involved in the modulation of a humoral response by lymphocytes and dendritic cells.

Following the clinical success of in vivo applications of therapeutic antibodies, CD22 was adopted as a target for antibody therapy and as a potential contributor to modulation of the immune response (Figure 4). Several features of the Siglec family members make them attractive for immunotherapy. Their selective expression by B lymphocytes coupled with their membrane dynamics, favoring a rapid endocytosis upon ligation by the antibody, make CD22 a good candidate for delivering cytotoxic hits. Further, their ability to modulate cellular signaling attributes to CD22 the role of an immunomodulator. Antibodies specific for human CD22 were designed to treat the majority of B-cell lymphomas and many types of ALL (vide infra). Other characteristics make CD22 more than a simple target. As a receptor rapidly internalized upon antibody ligation, CD22 can also be exploited as a carrier of a toxic payload operative in the cytoplasmic compartment. Consequently, antibody (or sialoside-based) immunotoxins can be used to target the tumor cell using CD22 as a means of entry [17]. 

The adverse reactions observed in vivo during anti-CD22 therapy prompted a revision of our understanding of the events taking place when an antibody reaches the target molecule. The anti-CD22 antibodies produced for therapy were designed by exploiting the specificity of variable regions and their interactions with the unique epitope. However, the direct specific ligation is also flanked by effects likely mediated by the antibody binding to complement fragments and to IgG Fc receptors (FcR). 

The molecular structure of the extracellular portion of human CD22 has recently been solved. This led to identification of an epitope recognized by the epratuzumab antibody [18]. The results confirmed that glycosylation of the target molecule might impair (and interfere with) the ability of the therapeutic antibody to access its epitope. The experience with different antibodies in in vivo therapy suggested that the events triggered by interactions taking place between the antibody and the target should be considered, particularly when the target acts as a receptor. A therapeutic antibody can be classified as agonistic, antagonistic, or indifferent when targeting a specific molecule induces a positive signal, or a signal blocking a receptor, or nothing, respectively [19]. The signals induced by antibody ligation to the target CD22 molecule have been widely studied. Less is known about the events linked to the modulation of CD22 expression and on the relevance of its localization in selected micro domains of the cell membrane [20]. Drawing on results obtained from different tumor models, it appears that the membrane micro domains specifically harboring the CD22 molecule are susceptible to the actions of cytokines, especially IL-4. Other observations indicate that CD22—in the same membrane domain—is functionally associated with CD32B (FcRII), with CD72 and paired immunoglobulin-like receptor-B ((PIR)-B) [21]. A common functional feature shared by these different molecules is their ability to negatively regulate BCR-mediated-signals. Further, they are also all susceptible to the action of IL-4, which reduces their expression on activated B lymphocytes, both at the level of messenger RNA and protein [22]. This reduced expression in response to continuous exposure to IL-4 is primarily mediated by Stat-6. Co-ligation of CD32B to BCR via intact IgG increases the threshold of activation and at the same time inhibits the ability of antigen recognition. From the translational perspective, it is relevant that IL-4 completely abolishes the negative signals mediated by CD32B. Similar effects were obtained by co-ligating CD22 with BCR. It may be that IL-4 enhances the B cell immune response by subtracting B lymphocytes from the suppression caused by inhibitory receptors. By coordinating the reduction in expression of inhibitory receptors and release from CD22 and FcRII-mediated inhibition, IL-4 might play a role in the T cell help of B cells and the development of T helper type 2 responses. Without IL-4, B cell activation would be more difficult to achieve, contributing to the maintenance of B cell tolerance in the absence of T cell help [23].

## 4. Therapeutic Anti-CD22 Antibodies in B-ALL

As is well known, CD22 is expressed by most blasts from the majority (60–90%) of B-cell malignancies. In the Inovate study, CD22 was expressed in >90% of B-ALL [24]. In a cohort of Italian B-ALL patients (142 individuals from different hematologic institutions; Lanza et al. 2019, unpublished data), CD22 expression was assessed as a percentage of positivity as well as a degree of positivity at disease onset and at several later points in time (remission, relapse, before and after HSCT). Only 2% of the ALL cases resulted CD22-negative (0–0.2% of positive blasts), 2% showed CD22 positivity in 1–10% of the blasts, 14% in 11–50% of the blasts, 16% in 51–90% of the blasts, and 55% in >90% of the blasts. These findings indicate that approximately 20% of B-ALL patients have <50% CD22^+^ blasts. Multi-parametric flow cytometry analysis of samples obtained from B-ALL patients at relapse or in partial remission showed a down-regulation or loss of the CD22 molecule by the surface of residual blasts in >10% of the cases. These observations may be of clinical utility in patients undergoing Inotuzumab ozogamicin (InO) therapy and for the assessment of MRD in ALL patients who have been treated with anti-CD22 antibodies. 

InO, a humanized anti-CD22 antibody conjugated to the cytotoxic antibiotic agent calicheamicin, was recently developed and tested in phase 1–2 and 3 clinical trials in the setting of B-cell malignancies [25]. Since most non-Hodgkin lymphomas (NHL) and B-ALL leukemic cells express surface CD22, a large number of preclinical and phase 1 studies evaluated the safety, antitumor activity, pharmacokinetics, and pharmacodynamics of InO for CD22^+^ B-cell tumors [26]. Initially, the antibody was developed for the treatment of NHL because of its activity in preclinical models and high response rates in indolent lymphomas. However, the results of a phase 3 trial were disappointing. For these reasons, the antibody was successively diverted to the treatment of CD22-positive ALL patients [27]. Preclinical studies using InO showed that it had strong in vitro activity and was capable of eradicating the disease in mice injected with different ALL cell lines. Preliminary testing in early-phase trials of human CD22^+^ ALL in humans demonstrated the feasibility and efficacy of the antibody treatment. Correlative analyses from an open-label, phase 1/2 study included analysis of the interrelation among InO pharmacokinetic exposure, hematologic measures and gene expression in response to treatment. After targeting CD22^+^ by InO in ALL blasts, preclinical and phase 1–2 studies showed that antibody ligation was rapidly followed by internalization of the complex into lysosomes, where calicheamicin is released. This drug binds to the DNA groove, leading to double-strand cleavage and subsequent apoptosis [28]. These studies also highlighted that the levels of unconjugated calicheamicin were below the limit of quantitation (50 pg/mL) for most patients and at different time points. Surface CD22 was observed as rapidly declining on lymphocytes, but the event was unrelated to InO concentration. Lymphocyte depletion from the blood was also rapid, and consistently observed regardless of the InO dose. CD22 was slowly re-expressed with a significantly different regeneration rate among patients. The percentage of BM blasts was directly related to the InO elimination rate, another point that led to the conclusions that (i) CD22 expression is not a significant determinant of InO concentration, and that (ii) the number of doses administered does influence the drug elimination rate, an effect considered to be linked to target-mediated drug clearance. These studies defined the steps through which target cells are killed by InO, namely: (i) successful delivery of the antibody/drug conjugate to the tumor microenvironment and (ii) antibody binding to surface CD22 followed by the internalization of the complex in the cytoplasmic district. The chemical linker is hydrolyzed, calicheamicin is activated by local thiols, and calicheamicin acts on DNA before its elimination by cellular efflux. Still unknown is which step is responsible for the polymorphic behavior and efficacy observed among ALL patients treated with InO. Drug efflux was not investigated in this setting; however, studies with gemtuzumab ozogamicin showed that an increased function of efflux pumps for calicheamicin reduced gemtuzumab efficacy [29,30,31]. Some of these factors are also included among mechanisms potentially driving resistance in B-ALL cells exposed to InO.

Importantly for a clinical perspective, phase 1–2 studies indicated that a weekly dose of InO (1.8 mg/m^2^ per cycle) is associated with limited toxicity and considerable clinical activity in patients with R/R ALL. For the above reasons, this dose of InO is the one of choice in ALL [25]. Findings from INO-VATE, a phase 3, open-label, randomized study, showed that patients who received InO had significantly higher CR rates (81% vs. 29%; *p* < 001), a lower disease burden during remission (78% vs. 28%, with BM blasts below the threshold for MRD), and more durable remission (median duration of remission 4.6 months vs. 3.1 months) compared with patients who received the investigator’s choice of standard chemotherapy [5]. The final INO-VATE results have recently been published and contain data obtained from a ≥2 year follow-up study as well as the patient characteristics associated with outcome. The median overall survival (OS) was 7.7 months for InO and 6.2 months for standard treatment, with two-year OS rates of 22.8% and 10.0%, respectively. The CR with incomplete hematologic recovery (CRi) rate was higher with InO than with SC (73.8% vs. 30.9%; *p* < 0001), with consistent CR/CRi rates among patient subgroups [32]. 

Other recent reports indicate that InO may also be effective in inducing a CR state in patients with MRD-positive disease. Taken together, these findings provide solid evidence that InO is highly efficient in B-ALL patients with R/R disease. Recently, several drugs targeting B cell-associated antigens (e.g., anti-CD20 (rituximab), CD19 (blinatumomab), and CD22 (inotuzumab)), have become available in Europe and USA, thus changing the treatment protocol for B-ALL patients. The combination of InO and low-intensity mini-HCVD (hyper CVAD) chemotherapy with (or without) blinatumomab are reported as conferring better outcomes than intensive salvage chemotherapy or InO alone [33] (Table 1). 

The occurrence of side effects related to the use of InO, the current clinical practice for its use, as well as a better designed application of allo-HSCT following InO therapy contributed to analysis of the efficacy and limits of InO in R/R ALL. Veno-occlusive liver disease (VOD) is a major adverse event (AE) associated with InO therapy, which is observed in 10–15% of the cases examined. Other important or serious AEs are tumor lysis syndrome, prolonged QT syndrome, infusion-related reactions, and hematological toxicity (neutropenia, febrile neutropenia and thrombocytopenia) [34]. A panel of expert hematologists and transplant physicians have summarized the recommendations for evaluation and management of the important AEs associated with InO, with special attention to diagnosis, prevention, monitoring and management of VOD. The interventions considered included prophylaxis medications, patient monitoring and assessment along with InO dose adjustment or discontinuation [35]. The application of these recommendations in our daily clinical practice, as well as the consolidated use of InO in ALL, has significantly reduced the frequency of severe and mild AEs after this type of therapy, both in the short- and in the long-term assessment after allo-HSCT. The main AEs associated with InO can be mitigated in clinics by adopting preventive measures and prompt diagnosis and management. Prophylactic pharmacologic agents are recommended in order to avoid VOD, while patients for whom HSCT is anticipated should limit their number of InO cycles to 2. After that, at least 4–6-week intervals should be left between InO infusion and beginning of the conditioning regimen for allo-HSCT. In the updated version of INO-VATE phase 3 trial, the frequency of VOD/sinusoidal obstruction syndrome (SOS) was 14%, still considerably more frequent than in the chemotherapy-treated group (2.1%). B-ALL patients at risk for VOD can be treated with ursodiol prophylactically. The pre-emptive use of defibrotide or its prophylactical use is under investigation; multiple clinical trials are addressing this point (Harmony, DefiFrance). A careful attention to fluid balance along with a daily monitoring of bilirubin, AST/ALT, renal function and abdominal volume are highly recommended for Inotuzumab-treated patients. Symptomatic care with diuretics, oxygen, and, hemodialysis/hemofiltration can be adopted for patients with suspected VOD. Paracentesis is recommended when ascites compromises respiration. Defibrotide is the only agent approved for treatment of VOD with renal or pulmonary impairment. The recommended dose is 6.25 mg/kg every 6 h for a minimum of 21 days; the therapy should be continued until the signs and symptoms of VOD resolve (up to a maximum of 60 days) [36,37]. 

A recent study also evaluated the quality of life (QoL) in ALL patients receiving InO. All patients completed the European Organization for Research and Treatment of Cancer (EORTC) QoL questionnaire and the EuroQoL 5 dimensions questionnaires at baseline, on day 1 of each cycle, and at the end of the treatment. The current patient-reported outcomes (PROs) data support the favorable benefit/risk ratio of InO for the treatment of relapsed/refractory ALL, with superior clinical efficacy and better QoL, as compared with SC [38]. 

## 5. B-ALL Subgroups 

Available data on Philadelphia-positive (Ph+) ALL, shows that CR rates are higher in patients treated with InO than in the SC group (73% vs. 15%), thus supporting the therapeutic role in this aggressive ALL disease. MRD negativity was achieved in 63% of Ph+ ALL treated with InO. Almost twice as many patients in the InO group proceeded to SCT after InO treatment as compared to SC. All these characteristics make InO an effective therapeutic tool in Ph+ ALL [7]. Although Ph+ patients treated with InO had a remission rate of 78.6%, Ph1 patients treated with SC had a better response rate (44.4% CR) than the other cytogenetic groups. Based on these premises, comparative analysis between InO and SC is apparently skewed by the improved efficacy of chemotherapy. A possibility is that the effect is secondary to a lack of prior exposure to chemotherapy in patients treated with TKIs, rather than a reduced response to InO. Further clinical studies in Ph+ ALL are needed before drawing definitive conclusions about the therapeutic role played by InO in this setting. The number of R/R ALL patients carrying t(4;11) and treated with InO is very small. However, the currently available results indicate that most of the subjects were resistant to InO treatment. It would be interesting to assess whether this refractoriness is attributable to an outgrowth of CD22^-^ blasts or to a lineage switch towards a myeloid phenotype. Calicheamicin sensitivity appeared to be less prominent in this genetic variant of ALL. 

The Inovate study did not included patients with a Ph-like signature; the consequence is that the role of inotuzumab in this setting remains unknown. However, more recent data from SWOG 1312 trial (a phase 1 study investigating inotuzumab in combination with cyclophosphamide, vincristine and prednisone for R/R CD22+ B-ALL) showed that 60% of patients with Ph-like signature achieved a CR state post-therapy (13 patients) [39]. The retrospective analysis from the Memorial Sloan Kettering Cancer Center has shown a 66% response rate (CR) in Ph-like genetics (two out of three patients treated). An observation of potential interest is that p53 mutated ALL were not associated with a worse response rate following inotuzumab treatment. These observations apparently show that molecular features associated with poor response to chemotherapy were not associated with inferior response rates and overall responses following inotuzumab. This cannot be the case for patients carrying the t(4;11) aberration [40].

In a recent single-arm, phase 2 study, InO in combination with low-intensity chemotherapy was used for old patients with Ph-negative ALL [28]. The results showed that InO is active in the disease, with a high proportion of patients achieving MRD negativity and improved survival outcomes. The safety profile was tolerable, with no treatment-related deaths [33].

## 6. Predictors of Response in ALL 

InO showed good results in most immunophenotypic and cytogenetic ALL patients with InO therapy, with the exception of t(4;11) patients. The pattern of CD22 expression on ALL blasts could be of clinical relevance in predicting disease response to InO. Adopting the cut-off value suggested by the INO-VATE study, 90% CD22 positivity on ALL cells was not a significant determinant for InO response (79.2% vs. 82.4%). This conclusion needs to be balanced out by simultaneous evaluation of the percentage of positivity, as well as the intensity of CD22 expression by ALL blasts [5]. According to our data, both CD22 surface expression levels and percentage of positivity display significant inter-patient variabilities. Indeed, the percentage of positive blasts may vary from 1% to 99%; at the same time, CD22 expression levels in single blast cells may vary from faint to very strong. These issues may be of clinical interest and at, the same time, may guide MRD detection following anti-CD22 therapy. It is a common notion that immune-targeted therapies (such as InO) induce rapid down-modulation of the target molecule. A possible solution suggested by the flow cytometrists is that of indicating alternative strategies that are not based on the analysis of CD22 expression by residual B-lymphocytes. Preliminary findings conducted in the context of blinatumomab therapy have led to the definition of alternative strategies to highlight residual disease in B-ALL after anti-CD22/anti-CD19 therapies [41]. Alternative strategies such as these will acquire relevance as the use of targeted immunotherapies becomes more widely available in clinical practice. We hold the view that CD22 expression in B-ALL blasts from patients undergoing InO treatment should be evaluated at different time points in order to assess the kinetics of disappearance and re-appearance of the molecule on residual B-ALL blasts. The sequential analysis of CD22 density on B-ALL blasts in patients responding (or not responding) to InO treatment may provide useful clinical and biological insights into these patients. Furthermore, patients had a higher rate of response during first salvage than in second salvage (87.7% vs. 66.7%). Age instead proved not to be a determining factor in response rate to InO, since patients in a wide age range (<55 or >55 years) had similarly high response rates to InO (80.3% vs. 81.4%). Further, the responses were superior to chemotherapy in both age groups (31.9% vs. 25%). Lastly, patients with larger marrow blasts infiltration experienced higher response rate if treated with InO, compared to standard treatments (86.7% vs. 77.9%) [38]. 

## 7. What Can Basic Science Add to Antibody Therapy 

The actual results with anti-CD22 antibodies were obtained in vivo either using naked IgG or as carriers of toxins or radionuclides. Another approach relied on glycans containing synthetic sialic acids used to target CD22. These ligands have an affinity sufficiently high to compete with the endogenous natural ligands: for this reason, they can be used to deliver toxins into target cells [42]. Previous experience with other targets in B lymphocytes (mainly, anti-CD20 antibodies optimized for clinical use) demonstrated that results obtained from basic science, especially in the field of FcRs, may find rapid applications in the design and use of a new generation of therapeutic antibodies with superior clinical performance. One of the starting points for improving the antibody was analysis of the interaction between the Fc region of an IgG and the specific Fc receptor. FcRs belong to a family of molecules that interact with specific domains located in the IgG Fc domain. In the context of antibody therapy, FcRs may acquire a role in the presentation of the therapeutic IgG to the target molecule expressed by the leukemic cells. Another contribution attributable to FcRs is their ability to transduce signals when expressed by effector cells or even by the target tumor. This aspect is relevant when the effector cells are endowed with a lytic potential or immune competence. Indeed, NK cells, T and B lymphocytes, monocytes/macrophages, and myeloid-derived suppressor cells (MDSC) have many of these receptors, although they are expressed with quantitative and qualitative differences. When an antibody reaches its target in an insoluble form (i.e., bound by a cell), its affinity and binding power improve significantly. Another aspect just beginning to be considered in therapy is that FcRs are signaling molecules that can deliver either positive or negative signals, according to the lineages where they are localized. A further important issue is that such signals may simultaneously be active on the effectors as well as on the target leukemic cells [43]. Another key issue that remains largely unexamined is linked to the chained events triggered by a simultaneous dual binding by the therapeutic antibody of the target molecule and at the same time by the engagement of the FcR localized on the same cell. This is defined as the scorpion effect: its functional consequences, especially in vivo, still need to be elucidated [44,45]. Neonatal FcR (FcRn) is another member of the family and a relative newcomer in the field, but it has attracted enormous attention in recent years [46]. Initially identified in maternal milk (for which it is named), its functions are mainly related to the homeostasis of IgG and B2-microglobulin, mostly through the kidneys. Experience with Daratumumab has shown that the life of the therapeutic antibody in vivo is longer than that of the normal IgG. For instance, Daratumumab lasts over 60 days, providing a therapeutic advantage [47]. One possibility is that this long persistence may be secondary to enhanced ligation by FcRs, including FcRn, which are characterized by a wide tissue distribution [48]. It is also possible that renal clearance of the therapeutic IgG is a factor that contributes to persistence. Pharmacokinetic evidence shows that the interaction between a therapeutic antibody and FcRn may significantly influence the life of the therapeutic reagent [49,50]. 

## 8. Alternative Approaches: CD22 CAR T-Cell Therapy in Refractory or Relapsed B-ALL 

Despite promising clinical outcomes worldwide after the introduction of CD19 CAR-T therapy, relapse after this approach is associated with a poor prognosis. CD22 CAR T-cell therapy was tested in 34 relapsed or refractory pediatric and adult B-ALL patients, who were unsuccessful with previous rounds of CD19 CAR T cell therapy [51]. CR or CR with incomplete count recovery (CRi) were achieved in 80% of the cases. Only a mild form of cytokine-release syndrome and neurotoxicity was observed in this sample. Seven CR patients received no further treatment, and three of them were still in remission at 6, 6.6, and 14 months after infusion. Eleven CR patients were promptly bridged to transplantation, and 8 of them were still in remission at 4.6–13.3 months after transplantation. The result was a one-year leukemia-free survival rate of 71.6%. 

The target CD22 was not lost nor were mutations observed among relapsed patients. Based on these findings, CD22 CAR-T therapy is a promising new tool for treating R/R B-ALL. In another study, donor-derived CAR T cells and CD19/CD22 dual-target CAR T cells were used in a clinical trial. Gene-edited “off-the-shelf” universal CAR T cells are also undergoing active clinical development [52]. 

## 9. Concluding Remarks

The CD22 cell surface molecule is expressed in most cases of B- ALL cells; however, its expression is variable in terms of percentage of positivity and numbers of molecule on individual leukemic blasts. These parameters may acquire relevance in longitudinal studies aimed at evaluating the expression of CD22 in different phases of the disease. The modifications may show clinical implications becoming instrumental in predicting the response to InO, the reference antibody currently used for the management of ALL. Furthermore, it is reasonable to expect that the presence of antigen-specific immune responses is associated with clinical responses to treatment. An implication of this is that patients having significant antibody and T cell responses against leukemia-associated proteins may also show better responses to inotuzumab therapy. Detailed assessment of CD22 antigens in B-ALL blasts obtained from different phases of the disease are likely to be helpful in improving the remission rate in patients undergoing anti-CD22 therapy. The loss of CD22 may be of clinical utility in CD22 CAR-T therapy. Moreover, improvements in CD22-based therapy may derive from implementation of biotechnological constructs enabling therapeutic antibodies to bind single or multiple molecules, leading to new ways of providing lytic effects [53].

## Figures and Tables

**Table 1:** Clinical trials of inotuzumab ozogamicin (InO) in B-ALL.

Study Phase	Disease	Intervention	Results	Reference
2	R/R Ph-Negative CD22 positive ALL	Mini-Hyper-CVD combined with InO and Rituximab	ORR was 78% (59% CR) MRD negative rates of 52% (at time of morphological response) and 82% (at three months). Median RFS of 8 months. Median OS of 11 months	[34]
3	R/R ALL	0.8 mg/m2 (D1), 0.5 mg/m2 (D8), 0.5 mg/m2 (D15) Versus Standard therapy	CR + CRi 80.7% (CR 35.8%). Median RFS of five months. Median OS of 7.7 months	[6]
1/2	R/R ALL	1.8 mg/m2 weekly	69% CR/CRi (29% CR)	[25]
2	B-ALL with positive MRD	InO	Recruiting	NCT03441061
4	R/R B-ALL	Investigating InO lower dose level (1.2 mg/m2/cycle) for those with higher risk for liver toxicity or VOD	Recruiting	NCT03677596
2	Precursor B-cell ALL in 56–74 years old	InO induction followed by conventional chemotherapy	Recruiting	NCT03460522
1/2	Ph + B-ALL and CML-blast phase	Bosutinib plus InO	Recruiting	NCT02311998
1	Acute leukemia of ambiguous lineage, Recurrent Ph + B-ALL, Recurrent Burkitt Lymphoma	Inotuzumab plus CVP (cyclophosphamide, Vincristine, Prednisone)	Recruiting	NCT01925131
2	Ph negative B-ALL	Inotuzumab followed by Blinatumomab	Recruiting	NCT03739814
1/2	R/R B-ALL	Inotuzumab plus Vincristine (liposomal)	Not yet recruiting	NCT03851081
2	Ph negative B-ALL in 55 years or older	InO plus CVP induction	Recruiting	NCT03249870
2	ALL with positive MRD prior to HSCT	InO	Not yet recruiting	NCT03610438
1/2	Untreated ALL in 60 years and older	InO plus combination chemotherapy	Recruiting	NCT01371630
3	Newly diagnosed B-ALL in 18–39 years old	InO plus chemotherapy	Recruiting	NCT03150693
2	R/R ALL	Lower dose InO	Recruiting	NCT03094611
2	ALL	InO plus Hyper-CVAD	Recruiting	NCT03488225
2	B-ALL in 1–21 years old	InO	Recruiting	NCT02981628
3	ALL	Tisagenlecleucel versus Blinatumomab or Inotuzumab	Not yet recruiting	NCT03628053

Abbreviations: R/R: refractory/relapsed; ORR: overall response rate; CR: complete remission; PFS: progression free survival; OS: overall survival; RFS: relapse free survival; MRD: minimal residual disease; CVAD: cyclophosphamide, vincristine, adriamycin, dexamethasone; ALL: acute lymphoblastic leukemia; CVP: cyclophosphamide, vincristine, prednisone; CVD: cyclophosphamide, vincristine, dexamethasone; VOD: veno-occlusive disease.
